# Business and property types experiencing excess violent crime: a micro-spatial analysis

**DOI:** 10.5249/jivr.v14i1.1566

**Published:** 2022-01

**Authors:** Daniel A. Bowen, Kurtis M. Anthony, Steven A. Sumner

**Affiliations:** ^*a*^ Division of Violence Prevention, National Center for Injury Prevention and Control, U.S. Centers for Disease Control and Prevention (CDC).

**Keywords:** Mapping, Violent Crime, Land Use, Pattern Theory, Crime Attractors

## Abstract

**Background:**

Beyond alcohol retail establishments, most business and property types receive limited attention in studies of violent crime. We sought to provide a comprehensive examination of which properties experience the most violent crime in a city and how that violence is distributed throughout a city.

**Methods:**

For a large urban city, we merged violent incident data from police reports with municipal tax assessor data from 2012-2017 and tabulated patterns of violent crime for 15 commercial and public property types. To describe outlier establishments, we calculated the proportion of individual parcels within each property-type that experienced more than 5 times the average number of crimes for that property-type and also mapped the 25 parcels with the highest number of violent incidents to explore what proportion of violent crime in these block groups were contributed by the outlier establishments.

**Results:**

While the hotel/lodging property-type experienced the highest number of violent crimes per parcel (2.72), each property-type had outlier establishments experiencing more than 5 times the average number of violent crimes per business. Twelve of 15 property-types (80%) had establishments with more than 10 times the mean number of violent incidents. The 25 parcels with the most violent crime comprised a wide variety of establishments, ranging from a shopping center, grocery store, gas station, motel, public park, vacant lot, public street, office building, transit station, hospital, pharmacy, school, community center, and movie theatre, and were distributed across the city. Eight of the 25 parcels with the highest amount of violent crime, accounted for 50% or more of the violent crime within a 400-meter buffer.

**Conclusions:**

All property-types had outlier establishments experiencing elevated counts of violent crimes. Furthermore, the 25 most violent properties in the city demonstrated remarkable diversity in property-type. Further studies assessing the risk of violent crime among additional property-types may aid in violence prevention.

## Introduction

Violent crime continues to be a leading public health problem nationally-annually there are over 19,000 homicides and over 1.5 million non-fatal assaults requiring medical treatment each year.^1^ To effectively prevent violent crime in a city, researchers and practitioners have asserted that it is often necessary to understand where violent crime is occurring and where victimization risk is greatest.^2,3^ To that end, geographically-focused crime research has progressively narrowed its attention to more precise levels of geography (i.e., from county to census tract to block group, and so on), with the most recent research seeking to understand micro-levels of geography.^4-7^ Micro-level analyses of crime have found that large differences in crime rates can occur between neighboring streets and between parks within cities.^4,8^

Researchers have also implemented micro-level analyses of crime to identify specific businesses and business-types that are at increased risk of violent crime. Due to the high number of violent crimes involving the use of alcohol, multiple studies have examined and established a positive association between increased alcohol outlet density and various types of violent crime, including assaults, robberies, and domestic violence.^9-14^ Some of these studies examine this association further by differentiating alcohol outlets as either "on-site" (e.g. bars and club) or "off-site" (e.g. liquor and package stores), which leads to increased utility in understanding the relationship between certain characteristics of an establishment and an increased risk of violence.^15,16^

To a lesser extent, other studies have examined the association of violent crimes in relation to other business-types, such as convenience stores, pawn shops, and fringe banking establishments.^17-20^ Studies related to these business-types suggest there is significant variability in the probability that any particular location will experience violent crime due to various environmental and business-specific characteristics.^3,21^ For example, convenience stores around a large metropolitan area in the U.S. were found to have a 77% increase in the odds of experiencing violent crime if they were surrounded by vacant land compared to those surrounded by open commercial land.^21^ Additionally, retail locations that operated for 24 hours had more than double the odds of experiencing violent injury compared with locations that do not operate 24 hours.^22^

While previous research has demonstrated that links exist between certain business-types and increased risk of violent crimes, it remains a challenge to comprehensively describe what individual businesses may be most at risk of experiencing violent crime across an entire city and many business-types have received limited attention in the research literature. One study aiming to more comprehensively explore land use, examined incidents of motor vehicle theft and assaults across all land use parcels in a suburb of Vancouver, Canada.^23^ This research detected a finding in that shopping centers experienced more assaults than bars.^23^ Other related research has examined how the proportion of certain land use types contributed to violent crime in sections of the city of Indianapolis, using 1000 square foot grid cells as the unit of analysis and exploring the interaction of certain features such as schools, hospitals, and cemeteries on violent crime.^24^ Nonetheless, the systematic patterns of violent crime across all commercial and public property in U.S. metropolitan areas remain under-examined.

Consequently, in this study we aimed to fully describe the distribution of violent crime by property type across an entire U.S. city by merging police-recorded incidents of violent crime with municipal tax-assessor data and boundary files. We postulated that this work would offer new insight into what locations experience the most violent crime and how that violent crime is distributed throughout a city. Better identifying property types where violent crime frequently occurs could help inform more effective resource allocation as well as inform city-level prevention strategies to reduce or prevent violent crime. 

## Methods 

**Violent Crime Data**

This study describes the distribution of violent interpersonal crime occurring in a large U.S. city, the City of Atlanta. This locale was selected for examination as it was part of the implementation of a place-based public health violence prevention strategy, however, no violence prevention efforts related to this strategy were underway in the study area.^25,26^ Data on violent crime were publicly available from the Atlanta Police Department (APD). Violent interpersonal crimes are defined as incidents of homicide, robbery, assault, or rape. Data on interpersonal violent crime incidents were gathered from official police reports, with the precise location of all interpersonal violent crimes geocoded by the APD and provided as exact coordinates without an offset. In this study, five years of data on violent crime from April 2012 to April 2017 were examined and all municipal parcels within the jurisdiction of the APD were included. 

**Property Type Data**

The aim of the study was to comprehensively describe the distribution of violent crime across all commercial and public municipal parcels in the City of Atlanta. Consequently, we obtained the publicly available 2016 county parcel shapefile from the County Board of Assessors. The parcel shapefile contained detailed geographic information on the borders of each parcel and the designated land use category of such parcels. We trimmed the shapefile to only include the 14,658 commercial and public parcels within Atlanta. Additionally, the County Board of Assessors classifies each parcel into one of 196 detailed commercial/public land use categories. The detailed land use categories were manually classified into 15 broader categories based on similar function and purpose of the establishments: Hotel/Lodging, Service Stations/Convenience Stores, Restaurants/Bars/Clubs, Recreation, Retail, Healthcare, Police/Fire/Correctional Facilities, Education, Government Owned, Religious, Transportation and Utilities (e.g., rail or bus terminal, airport, US Postal Service), Vacant Land/Abandoned Locations, Manufacturing/Industrial, Parking Lot/Garages, and Office/Financial. 

**Data Analysis**

To assess the frequency of violent crime by property type, we joined the municipal parcel shapefile with the dataset of geocoded violent incidents. Each violent incident located within a specified parcel was assigned to that parcel. The total number of violent crimes by property type was then tabulated using the 15 land use categories of interest. We also calculated the mean number of violent crimes per property type, the percentage of properties within a certain category that experienced a violent crime, and the percentage of violent crimes in the entire city contributed by that property type.

To explore the distribution of violent crime by each business/land use type we then calculated the number of violent crimes for each property in the dataset, arranged by property type. This allowed for examination of outliers among each category. For this study, we defined a parcel as being an outlier if it experienced five times the mean violent incidents experienced by other parcels within the same parcel category. Two and ten times the mean number of violent incidences were also examined to better understand the distribution of violent crime within parcel categories.

Lastly, to better understand the individual locations experiencing the largest amount of violent crime in the city, we further examined the 25 locations with the greatest frequency of violent crime over the study period. Specifically, we plotted the number of violent crimes at each location by type of violent crime (i.e., homicide, assault, rape, robbery). We also mapped the location of these 25 parcels to assess their geographic distribution across the study area. We overlaid these 25 parcels on a map of Census block groups in the APD jurisdiction showing the average number of crimes per parcel. We then calculated the total number of crimes in a 400-meter buffer around each of the 25 parcels to better understand their surrounding level of crime. Data management and analyses were conducted using R statistical software version 3.3.0 and ArcGIS 10.5.

## Results

Over the five-year study period, there were a reported 7,349 interpersonal violent crimes occurring on commercial/public parcels. Of these, 38.1% were aggravated assaults, 57.5% were robberies, 3.2% were rapes, and 1.2% were homicides. All incidents of interpersonal violent crime mapped to a parcel within the city.

Table 1 provides overall statistics on the 15 categories of land use and the distribution of violent crime across these categories. In total, there were 14,658 commercial and public parcels within APD’s jurisdiction, with 2,429 (16.6%) experiencing at least one violent crime over the study period. Hotels and lodging had the highest number of mean crimes per business (2.72), with 272 violent crimes occurring at 100 parcels. Service stations/Convenience stores and Restaurants/Bars/Clubs had the second and third highest mean number of violent crimes per business with 1.97 and 1.27, respectively. Retail locations, which experienced a mean of 1.06 violent crimes per business over the 5-year study period, contributed the greatest proportion of all violent crimes occurring in the city (27.8%) due to the large number of retail parcels in the city. Land classified as vacant or abandoned by the city tax assessor file contributed the second largest proportion of violent crime in the city, accounting for 14.8% of all violent crimes. 

**Table 1:** Frequency of Violent Crime by Parcel Category, 2012-2017, Atlanta, GA.

Parcel Category	Total Number of Crimes over Five Years (N = 7349)	Total Number of Parcels (N = 14658)	Mean Number of Crimes per Parcel	Number of Parcels with ≥ 1 Violent Crime (N = 2429)	Percentage of Parcels that experienced ≥ 1 Violent Crime	Percentage of Total Violent Crimes in City
Hotel/Lodging	272	100	2.72	62	62.0%	3.70%
Service Stations/Convenience Stores	1036	527	1.97	244	46.3%	14.10%
Restaurants/Bars/Clubs	599	472	1.27	217	46.0%	8.15%
Retail	2044	1920	1.06	625	32.6%	27.81%
Recreation	310	335	0.93	90	26.9%	4.22%
Healthcare	207	235	0.88	77	32.8%	2.82%
Police/Fire/Correctional Facilities	28	35	0.80	13	37.1%	0.38%
Education	243	492	0.49	104	21.1%	3.31%
Government Owned	458	1228	0.37	122	9.9%	6.23%
Religious	279	864	0.32	154	17.8%	3.80%
Transportation and Utilities	144	475	0.30	40	8.4%	1.96%
Office/Financial	291	1258	0.23	153	12.2%	3.96%
Vacant Land/Abandoned Locations	1088	4852	0.22	316	6.5%	14.80%
Manufacturing/Industrial	189	931	0.20	138	14.8%	2.57%
Parking Lot/Garages	161	934	0.17	74	7.9%	2.19%

Table 2 displays the proportion of establishments grouped by property type that experienced an elevated number of violent crimes. Although there is a strong right-skew to the data for each business type, each business type has a wide distribution of violent crime. Indeed, all land use categories have parcels with 5 or more times the mean number of violent crimes for that particular land use category. Furthermore, 12 of the 15 land use categories have businesses that experienced more than 10 times the average amount of violent crime for that particular land use category; this includes categories such as education (2.9%), healthcare (2.6%), and recreation (1.5%). Thus, extreme outliers of violent crime appear to be present among the majority of parcel/business-types. 

**Table 2:** Proportion of Outlier Establishments within Each Parcel Category that Experience an Elevated Number of Violent Crimes

	Proportion of Establishments Having a Violent Crime Count that is Greater than the Mean Count of Violent Crimes for Each Category					
	>2 times mean		>5 times mean		>10 times mean	
**Business Category**	n	%	n	%	n	%
**Hotel/Lodging (N = 100)**	13	13.0%	5	5.0%	0	0.0%
**Service Stations/Convenience Stores (N = 527)**	107	20.3%	24	4.6%	0	0.0%
**Restaurants/Bars/Clubs (N = 472)**	77	16.3%	20	4.2%	2	0.4%
**Retail (N = 1920)**	226	11.8%	98	5.1%	34	1.8%
**Recreation (N = 335)**	58	17.3%	20	6.0%	5	1.5%
**Healthcare (N = 235)**	38	16.2%	11	4.7%	6	2.6%
**Police/Fire/Correctional Facilities (N = 35)**	7	20.0%	2	5.7%	0	0.0%
**Education (N = 492)**	104	21.1%	24	4.9%	14	2.9%
**Government Owned (N = 1228)**	122	9.9%	47	3.8%	24	2.0%
**Religious (N = 864)**	154	17.8%	49	5.7%	14	1.6%
**Transportation and Utilities (N = 475)**	40	8.4%	17	3.6%	6	1.3%
**Vacant Land/Abandoned Locations (N = 4852)**	316	6.5%	127	2.6%	90	1.9%
**Manufacturing/Industrial (N = 931)**	138	14.8%	31	3.3%	11	1.2%
**Parking Lot/Garages (N = 934)**	74	7.9%	74	7.9%	28	3.0%
**Office/Financial (N = 1258)**	153	12.2%	61	4.9%	27	1.8%

**Figure 1:**
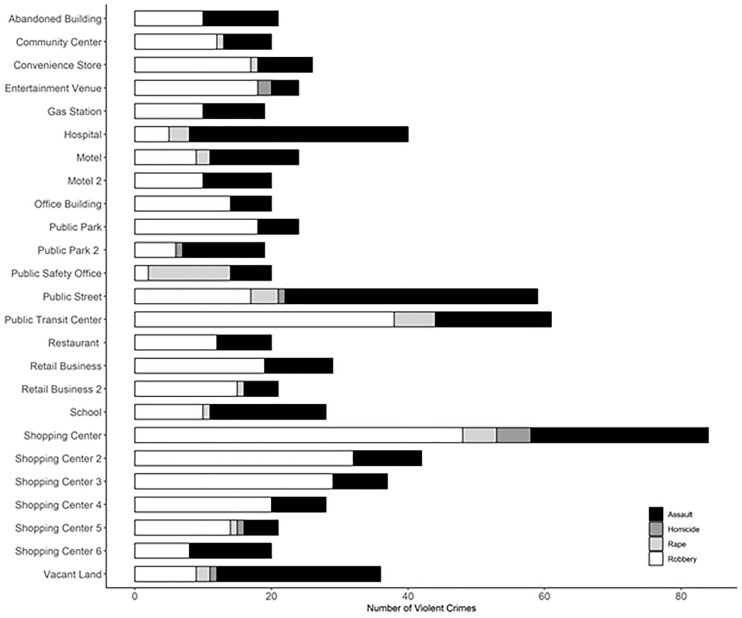
Characteristics of the 25 Parcels Experiencing the Highest Levels of Violent Crime, 2012-2017, Atlanta, GA

**Figure 2:**
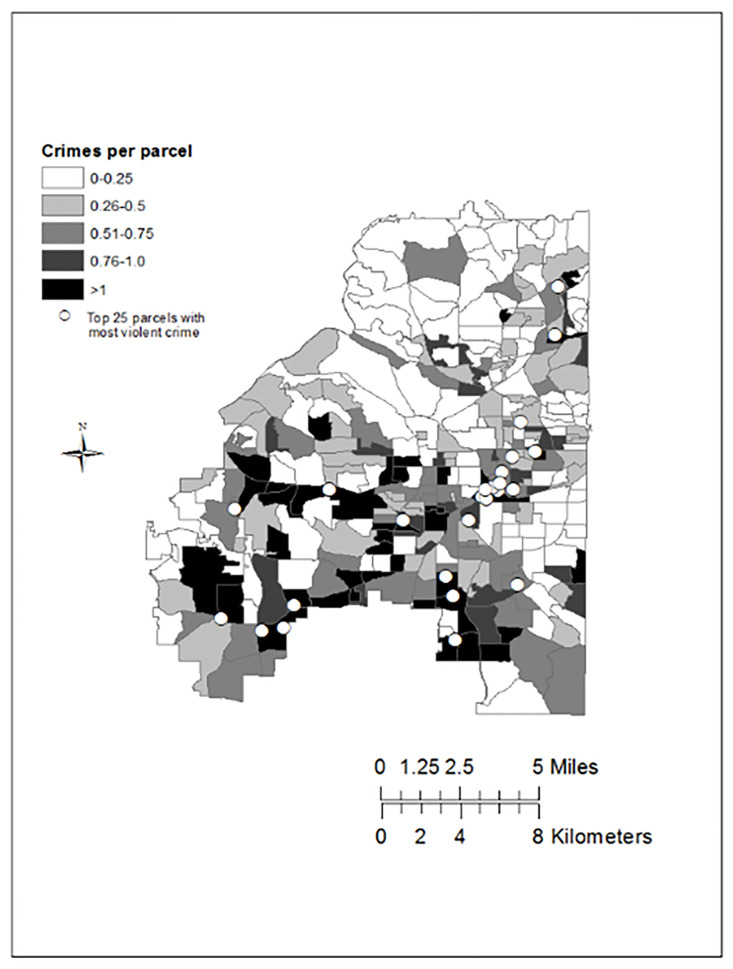
Geographic Distribution of the 25 Parcels Experiencing the Highest Levels of Violent Crime Footnote: Geographic subdivisions or boundary lines represent census block groups. White dots represent the location of the 25 parcels with the largest amount of violent crime across the entire city.

To examine the precise locations experiencing the greatest amount of violent crime in the city, Figure 1 examines the crimes committed at the 25 parcels that had the highest number of violent crime incidents over the study period, while Figure 2 is a map of the 25 parcels to examine both their geographic distribution across the study area and the mean number of violent crimes that occurred within each block group over the study period. Of these 25 top parcels (see Figure 1), 6 were shopping centers, 2 were stand-alone retail locations, 2 were motels, 2 were public parks, 2 were vacant properties, one was a convenience store, one was a public street, one was a city office building, and one was a public transit station. A hospital, a school, and a community center were also among the locations where the largest amount of violent crime occurred.

In general, robberies were the most common crime committed at the majority of the 25 locations (Figure 1). However, violent assaults comprised a large share of incidents at some of the top locations, such as the public street, vacant lot, school, and two motels. Examining the geographic distribution of the most violent parcels over the study period (Figure 2) revealed that these 25 properties were located in 19 unique census block groups. As expected, the most violent parcels were located in block groups with a relatively high level of crime. However, the 25 parcels differed with respect to the amount of crime in the surrounding 400-meter buffer. Eight of the 25 accounted for 50% or more of the violent crime within a 400-meter buffer (Table 3).

**Table 3:** Violent Crime Surrounding the 25 Parcels Experiencing the Highest Levels of Violent Crime, 2012-2017, Atlanta, GA.

Parcel	Number of Crimes at Parcel	Crime within 400-Meter Buffer	Parcels within Buffer	Percentage of crime within buffer occurring at parcel
Abandoned Building	21	101	120	20.8%
**Community Center**	**20**	**37**	**83**	**54.1%**
Convenience Store	26	213	400	12.2%
**Entertainment Venue**	**24**	**27**	**17**	**88.9%**
Gas Station	19	70	94	27.1%
Hospital	40	99	130	40.4%
Motel	24	77	73	31.2%
Motel 2	20	79	145	25.3%
Office Building	20	136	203	14.7%
**Public Park**	**24**	**24**	**5**	**100.0%**
Public Park 2	19	211	246	9.0%
Public Safety Office	20	221	215	9.0%
**Public Street**	**59**	**85**	**33**	**69.4%**
Public Transit Center	61	217	208	28.1%
Restaurant	20	260	250	7.7%
**Retail Business**	**29**	**57**	**26**	**50.9%**
Retail Business 2	21	113	46	18.6%
**School**	**28**	**40**	**37**	**70.0%**
Shopping Center	84	242	216	34.7%
**Shopping Center 2**	**42**	**62**	**43**	**67.7%**
Shopping Center 3	37	110	52	33.6%
Shopping Center 4	28	59	31	47.5%
Shopping Center 5	21	59	32	35.6%
Shopping Center 6	20	54	48	37.0%
**Vacant Land**	**36**	**53**	**20**	**67.9%**

Note: Parcels that experience 50% or more of the crime within a 400-meter buffer are in bold.

## Discussion

Our broad examination of violent crime across all city parcels revealed new insights; while several known relationships between violent crime and certain establishments were observed, additional property types not commonly examined in the literature were identified as having higher than expected counts of violent crime. Perhaps most importantly, we observed that all property types (whether previously identified in the literature to be at high risk for violent crime or not) had individual parcels that were marked outliers and experienced an excessive amount of violent crime, suggesting that all property types have problematic parcels.

The results of our study concur with known relationships between violent crime and establishments that sell alcohol (such as bars/restaurants/clubs) as well as convenience/service stores.^27,28^ However, more importantly, this study sheds light on additional property types not commonly examined in the literature. Interestingly, hotels and motels had the highest mean number of violent crimes per parcel, which may be partially explained by the high number of individual units that comprise a hotel/motel; however, office buildings, which similarly are comprised of a number of units, did not have a high mean number of crimes per parcel. Aside from a limited literature on prostitution and its occurrence at lodging establishments, there is scant literature on the patterns of violent crime at lodging businesses.^29-31^ Nonetheless, hotels/motels may serve not only as venues for sex work, but also for use of illicit substances as well as temporary lodging for transitory populations at higher risk of experiencing violent crime; this area of research merits further exploration. It is also notable that some property types, such as healthcare facilities, experienced increased levels of violent crime. Although workplace violence occurring in or around healthcare facilities has become a growing focus of investigation among medical researchers,^32,33^ there may be limited research into the importance of these locales as a component of a city’s overall pattern of violent crime. 

While understanding the distribution of violent crime by property type is important, notably, we detected that all business/property types had individual parcels that experienced an excess amount of violent crime compared to other parcels within the same property category. As stated previously, all property types had establishments experiencing more than 5-fold the mean number of violent crimes typical for that type of establishment and most even had a substantial proportion of establishments with greater than 10-fold the mean number of crimes. Research has identified that often a small number of establishments in a municipality are responsible for a disproportionate share of violent crime;^34^ our findings confirm this as well as highlight the diversity of establishments experiencing particularly excessive levels of violent crime. Indeed, though certain institutions, like recreation centers, are considered a beneficial community resource,^35^ it appears that even such institutions may have facilities that are associated with excess levels of violent crime. These findings also appear to show that all property types may have establishments that experience excess violent crime that may merit increased preventive attention. 

Among the 25 parcels with the most violent crime in the entire city, there was a wide variety of property types represented, such as a shopping center, grocery store, gas station, motel, public park, vacant lot, public street, office building, transit station, hospital, pharmacy, school, community center, and movie theatre. While the top 25 parcels tended to be located within block groups with higher-than-average violent crime, our analysis showed that a proportion of these parcels experience a majority, and in some cases all, of violent crime within their immediate vicinity. This result is interesting as it suggests that some parcels may be attractors to crime due to certain characteristics. Research in environmental criminology has developed theories that may aid future research on what specific characteristics of the parcels or surrounding neighborhoods explain the concentration of violent crime.^36,37^

This study has several limitations. First, although we examined data across a large group of land use categories, further work is needed to better understand the heterogeneity that may exist within each category and the association of such characteristics with excess levels of violent crime. Furthermore, the accuracy of the land use categories used are dependent on local governmental tax assessor records and classification. Misclassification bias may exist if errors in such municipal data are prevalent. It is also important to note that while a parcel can only be classified into one land use type, an establishment may have multiple functions which contribute to its risk of violence. For example, a hotel may also serve alcohol.^38^ Thus, further work is needed to elucidate the precise characteristics of high-risk establishments. Another limitation is the use of a single year of land use information in identifying parcels. It is possible that over the five years we examined, businesses changed within these parcels, especially as parcels became vacant or occupied. However, while some businesses could have changed, it is less likely that parcels changed in business type due to zoning and land use restrictions. It should also be noted that this study focuses on reported violent crime, which may underestimate total violence across the study area. Lastly, these results are focused on a single, large municipality in the southeastern United States. Other cities may exhibit unique patterns and the results herein may not be generalizable to all locales; however, the methodological approach could be applied broadly to other communities.

Nonetheless, this work carries important implications for violence prevention. While this study confirms the value of examining the occurrence of violent crime at a micro-spatial level,^4,39^ our findings also highlight the diversity of establishments experiencing excess levels of violence. An awareness of this diversity may aid in identifying and aiding such establishments with implementing place-based prevention approaches.^40^ Furthermore, this study demonstrates the potential of readily available municipal administrative data to conduct analyses that can guide prevention and policy decisions. Policy and program improvements might involve diverse strategies such as green space creation, enhanced lighting and visibility, adjustment of police patrols, income supports, policies to increase housing stability, or the creation of local business improvement districts.^41-45^ Comprehensive, systematic, and periodic examinations of properties experiencing excess levels of violent crime across an entire municipality may help guide violence prevention resources and improve the effectiveness of preventive efforts.

## References

[1] Centers for Disease Control Prevention National Center for Injury Prevention Control CfDCP. Web-based Injury Statistics Query and Reporting System (WISQARS). http://www.cdc.gov/ncipc/wisqars, accessed 4 March 2021.

[2] Eck JE, Weisburd DL (2015). Crime places in crime theory. Crime and place:. Crime prevention studies.

[3] Eck JE, Clarke RV, Guerette RT (2007). Risky facilities: Crime concentration in homogeneous sets of establishments and facilities. Crime Prevention Studies.

[4] Groff ER, Weisburd D, Yang S-M (2010). Is it important to examine crime trends at a local “micro” level?: a longitudinal analysis of street to street variability in crime trajectories. Journal of Quantitative Criminology.

[5] Weisburd D. The emergence of crime places in crime prevention. Punishment, Places and Perpetrators (From Punishment, Places and Perpetrators: Developments in Criminology and Criminal Justice Research). 2004;155-168.

[6] Harinam V (2020). Examining Micro-Level Homicide Patterns in Toronto, 1967 Through 2003. Canadian Journal of Criminology and Criminal Justice.

[7] Caplan JM, Neudecker CH, Kennedy LW, Barnum JD, Drawve G (2021). Tracking Risk for Crime Throughout the Day: An Examination of Jersey City Robberies. Criminal Justice Review.

[8] Groff E, McCord ES (2012). The role of neighborhood parks as crime generators. Security Journal.

[9] Livingston M (2011). A longitudinal analysis of alcohol outlet density and domestic violence. Addiction.

[10] Grubesic TH, Pridemore WA, Williams DA, Philip-Tabb L (2013). Alcohol outlet density and violence: the role of risky retailers and alcohol-related expenditures. Alcohol Alcohol.

[11] Grubesic TH, Pridemore WA (2011). Alcohol outlets and clusters of violence. Int J Health Geogr.

[12] Cunradi CB, Mair C, Ponicki W, Remer L (2011). Alcohol Outlets, Neighborhood Characteristics, and Intimate Partner Violence: Ecological Analysis of a California City. J Urban Health.

[13] Ratcliffe JH (2012). The spatial extent of criminogenic places: a changepoint regression of violence around bars. Geographical Analysis.

[14] Lardier DT, Reid RJ, Yu D, Garcia-Reid P (2020). A spatial analysis of alcohol outlet density and abandoned properties on violent crime in Paterson New Jersey. J Community Health.

[15] Snowden AJ, Pridemore WA (2014). Off-premise alcohol outlet characteristics and violence. Am J Drug Alcohol Abuse.

[16] Pridemore WA, Grubesic TH (2013). Alcohol Outlets and Community Levelsof Interpersonal Violence: Spatial Density, Outlet Type, and Seriousness of Assault. Journal of Research in Crime and Delinquency.

[17] Kubrin CE, Squires GD, Graves SM, Ousey GC (2011). Does fringe banking exacerbate neighborhood crime rates?. Criminology & Public Policy.

[18] Miles TJ. Markets for stolen property: Pawnshops and crime. Unpublished PhD Dissertation Chapter, University of Chicago Law School, Chicago, 2007.

[19] Amandus HE, Hendricks SA, Zahm D, Friedmann R, Block C, Wellford C (1997). Convenience store robberies in selected metropolitan areas: risk factors for employee injury. J Occup Environ Med.

[20] Reidy DE, Huntington C, Smith IV HW, Bogen KW, Estefan LF, Orchowski LM (2021). Community-level risk & protective correlates of violent crimes. Preventive Medicine.

[21] Hendricks SA, Landsittel DP, Amandus HE, Malcan J, Bell J (1999). A matched case-control study of convenience store robbery risk factors. J Occup Environ Med.

[22] Loomis D, Wolf SH, Runyan CW, Marshall SW, Butts JD (2001). Homicide on the job: workplace and community determinants. Am J Epidemiol.

[23] Kinney JB, Brantingham PL, Wuschke K, Kirk MG, Brantingham PJ (2008). Crime attractors, generators and detractors: land use and urban crime opportunities. Built Environment.

[24] Stucky TD, Ottensmann JR (2009). Land use and violent crime. Criminology.

[25] Mercer, Kollar LM, Sumner SA, Bartholow B, Wu DT, Moore JC, Mays EW (2020). Building capacity for injury prevention: a process evaluation of a replication of the Cardiff Violence Prevention Programme in the Southeastern USA. Inj Prev.

[26] Wu DT, Moore JC, Bowen DA, Mercer Kollar LM, Mays EW, Simon TR (2019). Proportion of Violent Injuries Unreported to Law Enforcement. JAMA Intern Med.

[27] Gorman DM, Speer PW, Gruenewald PJ, Labouvie EW (2001). Spatial dynamics of alcohol availability, neighborhood structure and violent crime. J Stud Alcohol.

[28] Toomey TL, Erickson DJ, Carlin BP, Lenk KM, Quick HS, Jones AM (2012). The association between density of alcohol establishments and violent crime within urban neighborhoods. Alcohol Clin Exp Res.

[29] Raphael J, Shapiro DL (2004). Violence in indoor and outdoor prostitution venues. Violence Against Women.

[30] Lowman J (2000). Violence and the outlaw status of (street) prostitution in Canada. Violence Against Women.

[31] LeBeau JL (2012). Sleeping with strangers: hotels and motels as crime attractors and crime generators. Patterns, Prevention, and Geometry of Crime. Routledge.

[32] Groenewold MR, Sarmiento RF, Vanoli K, Raudabaugh W, Nowlin S, Gomaa A (2018). Workplace violence injury in 106 US hospitals participating in the Occupational Health Safety Network (OHSN), 2012-2015. Am J Ind Med.

[33] Drummond DJ, Sparr LF, Gordon GH (1989). Hospital violence reduction among high-risk patients. JAMA.

[34] Sherman LW, Gartin PR, Buerger ME (1989). Hot spots of predatory crime: Routine activities and the criminology of place. Criminology.

[35] Peterson RD, Krivo LJ, Harris MA (2000). Disadvantage and neighborhood violent crime: Do local institutions matter?. Journal of Research in Crime and Delinquency.

[36] Brantingham PL, Brantingham PJ (1993). . Environment, routine and situation: Toward a pattern theory of crime. Advances in Criminological Theory.

[37] Brantingham P (2013). Crime pattern theory. Environmental criminology and crime analysis.. Willan.

[38] Sanchez-Ramirez DC, Voaklander D (2018). The impact of policies regulating alcohol trading hours and days on specific alcohol-related harms: a systematic review. Inj Prev.

[39] Nelson AL, Bromley RD, Thomas CJ (2001). Identifying micro-spatial and temporal patterns of violent crime and disorder in the British city centre. Applied Geography.

[40] Shepherd JP, Sumner SA (2017). Policing and Public Health—Strategies for Collaboration. JAMA.

[41] Florence C, Shepherd J, Brennan I, Simon T (2011). Effectiveness of anonymised information sharing and use in health service, police, and local government partnership for preventing violence related injury: experimental study and time series analysis. BMJ.

[42] Garvin EC, Cannuscio CC, Branas CC (2013). Greening vacant lots to reduce violent crime: a randomised controlled trial. Inj Prev.

[43] Carter SP, Carter SL, Dannenberg AL (2003). Zoning out crime and improving community health in Sarasota, Florida: “crime prevention through environmental design”.. Am J Public Health.

[44] MacDonald J, Golinelli D, Stokes RJ, Bluthenthal R (2010). The effect of business improvement districts on the incidence of violent crimes. Inj Prev.

[45] Jacoby SF, Kollar LMM, Ridgeway G, Sumner SA (2018). Health system and law enforcement synergies for injury surveillance, control and prevention: a scoping review. Inj Prev.

